# A Multi-Stage Feature Aggregation and Structure Awareness Network for Concrete Bridge Crack Detection

**DOI:** 10.3390/s24051542

**Published:** 2024-02-28

**Authors:** Erhu Zhang, Tao Jiang, Jinghong Duan

**Affiliations:** 1 Department of Information Science, Xi’an University of Technology, Xi’an 710048, China; 2210821086@stu.xaut.edu.cn; 2 School of Computer Science and Engineering, Xi’an University of Technology, Xi’an 710048, China; jh-duan@xaut.edu.cn

**Keywords:** concrete bridge crack detection, structure awareness, feature attention fusion, multi-stage feature aggregation, strip convolution

## Abstract

One of the most significant problems affecting a concrete bridge’s safety is cracks. However, detecting concrete bridge cracks is still challenging due to their slender nature, low contrast, and background interference. The existing convolutional methods with square kernels struggle to capture crack features effectively, fail to perceive the long-range dependencies between crack regions, and have weak suppression ability for background noises, leading to low detection precision of bridge cracks. To address this problem, a multi-stage feature aggregation and structure awareness network (MFSA-Net) for pixel-level concrete bridge crack detection is proposed in this paper. Specifically, in the coding stage, a structure-aware convolution block is proposed by combining square convolution with strip convolution to perceive the linear structure of concrete bridge cracks. Square convolution is used to capture detailed local information. In contrast, strip convolution is employed to interact with the local features to establish the long-range dependence relationship between discrete crack regions. Unlike the self-attention mechanism, strip convolution also suppresses background interference near crack regions. Meanwhile, the feature attention fusion block is presented for fusing features from the encoder and decoder at the same stage, which can sharpen the edges of concrete bridge cracks. In order to fully utilize the shallow detail features and deep semantic features, the features from different stages are aggregated to obtain fine-grained segmentation results. The proposed MFSA-Net was trained and evaluated on the publicly available concrete bridge crack dataset and achieved average results of 73.74%, 77.04%, 75.30%, and 60.48% for precision, recall, F1 score, and IoU, respectively, on three typical sub-datasets, thus showing optimal performance in comparison with other existing methods. MFSA-Net also gained optimal performance on two publicly available concrete pavement crack datasets, thereby indicating its adaptability to crack detection across diverse scenarios.

## 1. Introduction

As an important transportation infrastructure, the safety of bridges is crucial to people’s social lives and economic activities [1]. The main objective of structural health monitoring (SHM) of concrete bridges is to ensure their safety, reliability, and long-term performance. Thus, the monitoring of the structural conditions of bridges is essential. When performing SHM, a variety of factors need to be considered that have the potential to affect the structural integrity and service life of bridges, such as crack and damage monitoring, corrosion assessment, load and stress analysis, material property degradation, the influence of environmental factors (e.g., wind, temperature, humidity, etc.), and vibration characterization [1]. While cracks are one of the most common bridge defects and the earliest sign of bridge surface deterioration [2], timely detection and repair of cracks would be beneficial to greatly reduce bridge maintenance costs and avoid disastrous consequences. In the early days, crack detection on bridges relied heavily on manual labor using measuring tools and the human eye. This method was difficult to operate, had a high risk factor, and was highly subjective. The use of computer vision has made it common practice to capture images of every part of a bridge structure using a robotic arm or drone equipped with a camera. The data are then processed to detect cracks. However, detecting bridge cracks is still challenging due to their thin and long shape, low contrast between cracks and backgrounds, and many noisy interferences.

In previous studies, many traditional methods have been proposed for crack detection [3,4,5,6,7,8]. The traditional methods mainly include edge detection [3,4], threshold segmentation [5,6], and machine learning [7,8,9,10,11,12] methods. The methods based on edge detection and threshold segmentation are sensitive to background noise interference and reduce the precision of crack detection under complex backgrounds. While approaches based on machine learning are used to improve the effectiveness of crack detection by selecting expertly handcrafted features, [10] proposed a road crack detection algorithm based on scale invariant feature transform (SIFT) and backpropagation (BP) neural networks. SIFT is used to extract the feature point information of the crack image, and then a BP neural network is used for training and identification. The authors of [11] proposed a method for detecting concrete surface cracks using the histogram of oriented gradients (HOG). HOG features identify cracks by analyzing the direction and intensity of local gradients in the image, which has the advantages of high computational efficiency and insensitivity to changes in illumination. The authors of [12] proposed a crack detection method based on random structured forest, which utilizes integral channel features to capture the inherent structured information of cracks and combines this representation with random structured forest to generate a crack detector capable of identifying complex cracks. These methods have proven to be effective in detecting cracks with high contrast, a single shape, and a clear textured background. However, these methods struggle to extract robust features under various conditions and cannot be adapted for crack detection in different environments.

In recent years, deep learning techniques have gained widespread popularity and demonstrated powerful performance in image classification, object detection, and image segmentation [9,13] and have been widely applied to crack detection. Many previous studies [14,15,16,17,18] mainly used segmentation networks (e.g., FCN [15], UNet [16], SegNet [17], etc.) for pixel-level crack detection, and the crack detection precision of these approaches was greatly improved compared with traditional approaches. However, these approaches are poorly adapted to crack detection with complex background noise. Subsequently, numerous crack detection methods [19,20,21,22,23,24,25,26,27,28] have been proposed to further enhance the capability of crack detection, focusing on three major aspects: enlarging the receptive field, fusing multi-scale features, and adopting attention mechanisms. For example, [2,19,21,27] used dilated convolutions to increase the receptive field. In [20,21,22,25,26], the authors constructed a feature pyramid to obtain multi-scale features and leverage deep supervision learning. The authors of [22,23,24,28] introduced attention mechanisms to emphasize the semantic features of cracks. All of these approaches have led to a certain improvement in the precision of crack detection, but they are mainly for detecting pavement cracks. When they are migrated to detect bridge cracks, the detection performance drastically reduces due to the slender nature of bridge cracks; the low contrast between cracks and backgrounds; and the presence of various interfering factors, such as mud stains and water stains. Therefore, detecting bridge cracks in real-life scenarios is still challenging. Previous CNN-based crack detection methods did not consider the slender nature of bridge cracks and were strongly affected by noisy backgrounds, resulting in limited capability to simultaneously capture global and local crack features. Consequently, the overall crack detection performance of these methods is compromised, especially for the detection of fine-grained cracks.

In this work, a multi-stage feature aggregation and structure awareness network for bridge crack detection is proposed. Because square convolution kernels are employed in most CNN architectures, they are not suitable for capturing the linear features of cracks. However, strip convolution is more concerned with the shape of cracks. Inspired by this, a structure-aware convolution block is proposed in MFSA-Net by integrating square convolution with strip convolution, which can perceive the linear structure of cracks. To further sharpen the edges of bridge cracks and suppress interference from irrelevant background regions, this paper proposes a feature attention fusion block to fuse features from the encoder and decoder at the same stage. In the crack detection stage, the features from different stages are aggregated to form a fine-grained segmentation map. In the training stage, due to the impact of batch size on training results and the limited memory resources, group normalization (GN) [29] is also used as an alternative to batch normalization (BN) for the normalization layer in MFSA-Net. In brief, the proposed MFSA-Net has the advantages of capturing the linear features of cracks, fusing local detailed features and global semantic features of cracks, and establishing long-range dependencies between discrete crack regions. The main contributions of this paper are summarized as follows:

(1) A multi-stage feature aggregation and structure awareness network is proposed for bridge crack detection. The proposed MFSA-Net can effectively perceive the elongated structure of bridge cracks and obtain fine-grained segmentation results in a multi-stage aggregation manner.
(2) A structure-aware convolution block (SAB) is proposed, where the square convolution can extract local detailed information and the strip convolution is employed to refine the thin and long features of cracks for establishing long-range dependencies between discrete regions of cracks.
(3) A feature attention fusion block (FAB) is designed for fusing local context information and global context information with the attention mask, which can suppress interference from irrelevant background regions and sharpen the edges of bridge cracks.

Compared with the traditional models, strip convolution can fit the linear structural features of cracks, capture the global features, and also suppress background interference. The traditional models mainly use CNN architecture, which cannot fit the linear structural features of cracks well. It also increases the receptive field by increasing the convolution kernel to obtain global information, which introduces more background interference for crack detection.

## 2. Related Works

### 2.1. Crack Segmentation

Since Yang et al. [15] used FCN networks for pixel-level crack detection, many semantic segmentation models (e.g., SegNet [17], U-Net [16], and DeepLab [18]) have been employed for pixel-level crack detection. Han et al. [30] proposed a skip-level round-trip sampling block to improve the pooling and upsampling methods of U-Net, which can combine the properties of different receptive fields. Lin et al. [31] used a full-attention strategy in U-Net, combining the attention mechanism and the output of skip connections at each coding layer. These approaches did not take into account the imbalance between foreground and background pixel samples in crack segmentation. Li et al. [32] proposed a combination of pixel-based adaptive weighted cross-entropy loss and Jaccard distance based on U-Net to reduce the imbalance between cracked and non-cracked pixels in crack images. The authors of [33,34] captured global contextual information using dilated convolution blocks to expand the receptive field. Multiple dilation (MD) blocks were proposed in [21,27] to extract crack features with multiple context sizes and detect cracks of different widths and topological structures. The authors of [2,19] utilized hybrid dilated convolution blocks to alleviate the grid effect caused by dilated convolutions. In [20,25,26], the authors employed hierarchical multi-scale feature fusion to integrate contextual information into low-level features for crack detection and used deep supervision to take advantage of feature information from different scales. The method can combine high-level and low-level semantic information for accurately detecting or segmenting the object. Chen et al. [35] incorporated the rotational invariance property of cracks and introduced active rotational filters (ARF) [36] to encode the rotation invariance into the network. While these segmentation-based crack detection methods have shown promising results, they still fall short of satisfactory performance in terms of pixel-level segmentation precision and lead to blurry and coarse segmentation results.

### 2.2. Attention Mechanisms

To make the network focus more on the semantic features of cracks while suppressing non-semantic features, [21,34,37] introduced attention mechanisms into the network to pay more attention to the semantic information of cracks. Due to the excellent performance of transformers [38] in modeling long-range dependencies, Zhang et al. [24] proposed the UTCD-Net model for dam crack detection, which utilizes a dual-branch structure to fuse the global features extracted by the transformer branch and the local features extracted by CNN via the fusion module. Liu et al. [23] proposed a fine-grained crack detection network, CrackFormer, using a self-attention module to construct the network and extract global features of cracks. Xu et al. [39] proposed a locally enhanced transformer network (LETNet) to completely and efficiently detect road cracks. Transformer is employed to model long-range dependencies and compensate for low-level and high-level local features by designing a convolution stem and a local enhancement module. The squeeze-and-excitation (SE) [40] module uses global average pooling and a linear layer to calculate a scaling factor for each channel and then scales the channels accordingly. Visual attention (VAN) [41] decomposes the large convolution kernel into spatial depth convolution, spatial depth dilated convolution, and pointwise convolution, which addresses the increased computational cost caused by enlarging the convolution kernel and replaces self-attention with large kernel attention. The introduction of attention modules aims to model long-range dependencies and capture global contextual information. However, these approaches introduce more noise interference information while modeling long-range dependencies between crack regions.

### 2.3. Strip Convolution

The authors of [42] proposed strip pooling (SPNet). Different from traditional spatial pooling, this method considers a long but narrow kernel, i.e., 1 × N or N × 1. As a result, it can capture long-range dependencies between discretely distributed regions and improve the network’s effectiveness at segmenting strip objects. SegNeXt [43] decomposes a large convolution kernel into strip convolution in two directions by employing multiple large kernels to extract and fuse multi-scale information. The authors of the work demonstrated that strip convolution is effective in detecting strip-like objects, such as humans and telephone poles, in the segmentation scenes. The authors of [44,45] employed strip convolution for road extraction from aerial and satellite imagery. Because roads have elongated features, strip convolution is more consistent with the shape of roads and is able to extract strip-like features. SpinNet [46] used strip convolution for lane line detection and extracted the linear features of lane lines from different directions by rotating the feature maps. HCSCNet [47] involves a hierarchical correlation strip convolution network for text recognition that extracts narrow stroke features in text using strip convolution. These methods have demonstrated that strip convolution can extract long-term and narrow linear features and effectively capture long-range contextual information, which is suitable for the detection of objects with long strip-like features. As bridge cracks typically have long strip-like characteristics, we introduced strip convolution into the bridge crack detection network and captured the direction information of bridge cracks through strip convolution in four directions.

## 3. The Proposed Method

### 3.1. Network Architecture

**Figure 1:**
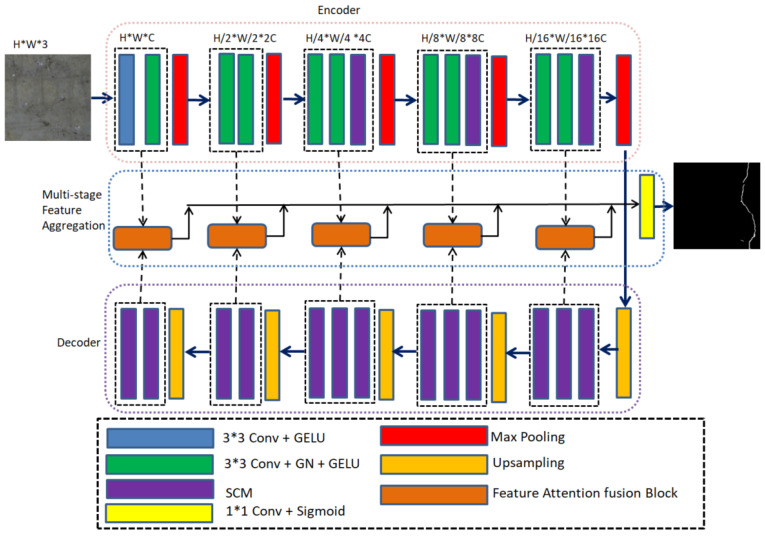
The architecture of the proposed MFSA-Net.

As shown in Figure 1, the overall architecture of the proposed MFSA-Net is an encoder–decoder structure similar to that of CrackFormer [23]. Unlike [23], the encoder is carefully designed in a hybrid convolution manner, where a structure-aware convolution block (SAB) is proposed. CrackFormer primarily utilizes self-attention to construct the encoder, enabling it to model the long-distance dependence of cracks. However, it ignores the local features of cracks. This paper proposes the use of strip convolution to model the long-distance dependence of cracks according to the long and thin characteristics of cracks and suppress the background interference near the crack regions while maintaining the convolution operation and obtaining the local features.

The proposed SAB is constructed by a traditional 3 × 3 convolution and a strip convolution, where the strip convolution is employed to efficiently capture the elongated features of cracks. On the decoder side, strip convolution is employed exclusively, which is able to recover the global features of cracks in a phased manner and establish the long-term dependencies between crack regions. The multi-stage feature aggregation module uses the proposed feature attention fusion block to fuse the local and global context features of cracks and then aggregate features from different stages to generate the final fine-grained segmentation results.

### 3.2. Encoder

Currently, most CNN network architectures usually use square convolution kernels to extract features within a local square window. For general semantic segmentation scenarios, most of the detection subjects are natural objects with chunk shapes, so the conventional square convolution can achieve great detection results. However, cracks have characteristics such as being long but narrow, having a large span, etc., which means the square convolution is not able to capture the linear features of cracks well and model long-distance dependencies between discretely distributed regions of cracks. On the contrary, strip convolution uses a long but narrow convolution kernel shape, which is more consistent with the shape of bridge cracks. Thus, it is easy to capture the enlarged features of cracks and model long-range dependencies between regions with discrete distributions of bridge cracks. In view of this, a structure-aware convolution block is proposed in MFSA-Net. As shown in Figure 1, the last three stages of the encoder are constructed by the proposed SAB. The SAB consists of two square convolution modules and one strip convolution module (SCM), where the square convolution is used to extract local detailed information and the strip convolution is employed to refine the enlarged features of cracks.

**Figure 2:**
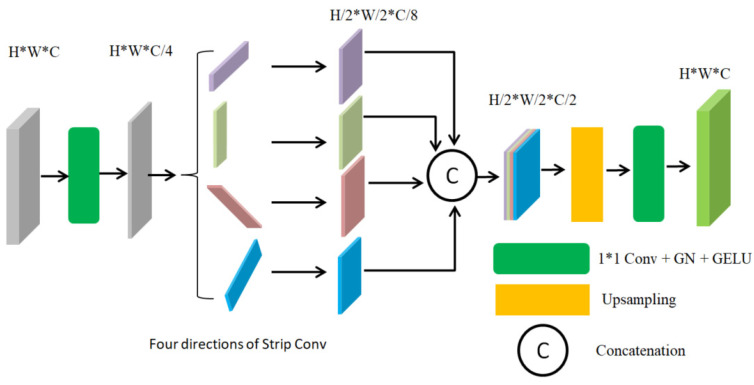
The strip convolution module.

Different from ordinary convolution modules, the SCM [45] can utilize multidirectional strip convolution to capture long-range context information from different directions. In this paper, strip convolution in four directions, namely, horizontal, vertical, left diagonal, and right diagonal, is used to construct the strip convolution module, as shown in Figure 2. Let $X\in {\mathbb{R}}^{H\times W\times C}$ denote the input tensor of the SCM, where H, W, and C are the height, width, and number of channels of the input tensor, respectively. In order to keep the total number of network parameters and computational cost constant, X is first reduced to C/4 channels after a 1 × 1 convolution. Then, it is fed into four parallel strip convolutions of different orientations for feature extraction, and the extracted features are concatenated. Finally, the feature map size and the number of channels output from the SCM are adjusted by upsampling and a 1 × 1 convolution.

Let $w\in {\mathbb{R}}^{2k+1}$ denote the strip convolution filter of size 2k+1 and $y_{D}\in {\mathbb{R}}^{H\times W\times C^{\prime }}$ denote the output result of strip convolution. $x\in {\mathbb{R}}^{H\times W\times C}$ is the input to the strip convolution, and $D=(D_{h},D_{w})$ represents the direction of the filter w. Then, the strip convolution can be formulated as Equation (1):

(1) $$y_{D}[i,j]={\left(x*w\right)}_{D}[i,j]=\sum_{l=-k}^{k}x[i+D_{h}l,j+D_{w}l]\cdot w[k-l],$$

where x∗w denotes the strip convolution operation; *D* is the direction vector of the strip convolution; and the direction vector $\left(D_{h},D_{v}\right)$ is (0, 1), (1, 0), (1, 1), and (−1, 1) for horizontal, vertical, left diagonal, and right diagonal strip convolutions, respectively. For the filter w, we set k=4 to make each strip convolution have 9 parameters, which is the same as a 3 × 3 convolution filter. Instead of a 3 × 3 convolution, a 4-direction parallel strip convolution is used in the strip convolution module. The four directions in the strip convolution are consistent with the distribution of most cracks in bridge crack images and are relatively easy to implement.

As shown in Figure 1, the encoder consists of five stages. The 1st stage consists of two ordinary convolutional layers (3 × 3 Conv-GELU and 3 × 3 Conv-GN-GELU) and one maximum pooling layer. The 2nd stage consists of two convolutional layers (3 × 3 Conv-GN-GELU) and one maximum pooling layer. To enlarge the effective receptive field of the network [44] and perceive the slender nature of bridge cracks, the third, fourth, and fifth stages were constructed by the SAB. Specifically, the SAB is composed of two ordinary convolutions (3 × 3 Conv-GN-GELU), one SCM (Strip Conv-GN-GELU), and one maximum pooling layer. For the maximum pooling layer, 2× downsampling is performed using a 2 × 2 window and a stride of 2 is used to obtain multi-scale feature maps.

It is worth noting that, due to memory consumption constraints, the GN layer is used instead of the BN layer in the proposed MFSA-Net. This is because when the batch size becomes smaller, it is easy to cause inaccurate batch statistic estimation, which leads to an increase in the BN’s error. Moreover, the GN layer divides the channels into groups and computes the mean and variance for normalization within each group, so the GN layer is not affected by batch size, and its accuracy is stable over a wide range of batch sizes.

### 3.3. Decoder

As shown in Figure 1, the decoder also consists of five stages, each of which is composed of an upsampling operation and two or three SCMs. The decoder is built using SCM, which is capable of extracting the long and narrow features of cracks, modeling the long-distance dependencies between isolated regions of cracks from multiple directions, and capturing the global contextual information of cracks. In particular, at each stage, the feature map is first upsampled by a factor of 2 using bi-linear interpolation, while the feature dimension is reduced by half. Then, the crack features are purified using three SCMs to establish the regional relationships between different cracks.

### 3.4. Multi-Stage Feature Aggregation

To fully utilize the local features in the encoder while retaining the global features in the decoder, a feature attention fusion block is proposed, which can sharpen crack edges and suppress interference from non-crack regions. As shown in Figure 1, there are five stages in the encoder and decoder parts. Therefore, a multi-stage feature aggregation strategy is used to form the final crack segmentation results. Specifically, at each stage, the FAB is used to fuse the local features extracted from the encoder with the global features extracted from the decoder. Then, the fused features of each stage are upsampled to the same size as the original image, and the segmentation mask is obtained through a 3 × 3 convolution to form the prediction segmentation map of each stage. Finally, the prediction segmentation maps of the five stages are concatenated together and passed through a 1 × 1 convolution to obtain the final fine-grained segmentation mask.

**Figure 3:**
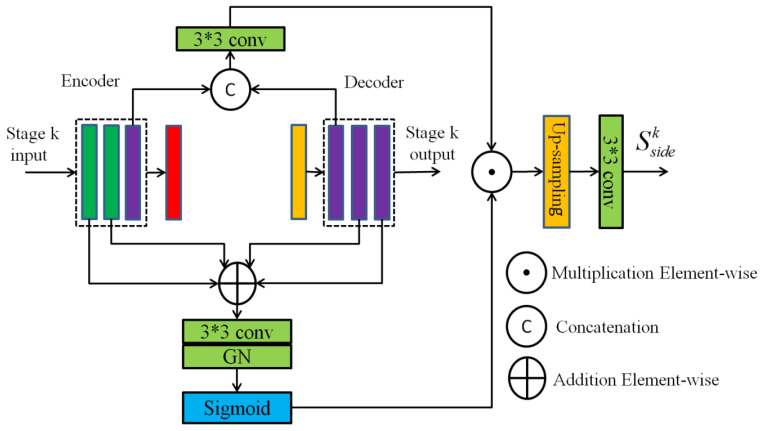
The feature attention fusion block.

As shown in Figure 3, the FAB first forms an attention mask using the local features in the encoder and the global features in the decoder, which makes it possible to highlight the local detail information of the cracks as well as focus on the global information of the cracks. This attention is then applied to the features formed by the concatenation of the encoder and decoder to activate crack features and suppress the non-crack ones for the purpose of sharpening the crack edges. Further, the fused features are sampled to the input image size by upsampling and converted into a crack segmentation prediction map using a 3 × 3 convolution. Finally, the segmentation prediction maps at each stage are concatenated to output the final crack segmentation map by a 1 × 1 convolution.

The *k*th stage is taken as an example to describe how the feature attention fusion block performs feature fusion to form the segmentation map. As shown in Figure 3, let ${\{X}_{1}^{k}{,X}_{2}^{k}{,X}_{3}^{k}\}$ and $\left\{Y_{1}^{k},Y_{2}^{k},Y_{3}^{k}\right\}$ be the feature maps from the encoder and decoder, respectively. Then, the generated attention mask map $A_{mask}^{k}$ is shown in Equation (2):

(2) $$A_{mask}^{k}=\sigma (GN({⊗}_{3\times 3}⊕(X_{1}^{k},X_{2}^{k},Y_{2}^{k},Y_{3}^{k}))),$$

where ⊕(⋅) denotes the element-wise addition of the tensor, ${⊗}_{3\times 3}$ represents a 3 × 3 convolution operation, GN(•) means group normalization, and σ(⋅) is a Sigmoid activation function.

Next, the side output $S_{side}^{k}$ of the *k*th stage is formed by the attention mask map and skip connection, as shown in Equation (3):

(3) $$S_{side}^{k}={⊗}_{3\times 3}(Up_{H\times W}(A_{mask}^{k}⊙({⊗}_{3\times 3}\Gamma (X_{3}^{k},Y_{1}^{k})))),$$

where Γ(⋅) denotes a tensor concatenation operation, ⊙ represents an element-wise multiplication operation., and $Up_{H\times W}(\cdot )$ denotes the upsampling to the input image size. Through upsampling the features of each stage to the input image size, the predicted segmentation result is obtained through a 3 × 3 convolutional layer. In this way, five predicted results, $S_{side}^{k},k=1,2,\ldots ,5$, can be obtained.

Finally, the predicted results of all stages are concatenated together and fused by a 1 × 1 convolution to generate the final fine-grained segmentation result $S_{fuse}$, as shown in Equation (4). Similar to FPHBN [26], DeepCrack [20] and HCNN [25], etc., all side and fused outputs are supervised learning conducted from the crack ground truth labels using deep supervision.

(4) $$S_{fuse}=\sigma ({⊗}_{1\times 1}\Gamma (S_{side}^{1},S_{side}^{2},S_{side}^{3},S_{side}^{4},S_{side}^{5}))$$

### 3.5. Loss Function

In bridge crack segmentation, because the number of pixels in the cracks is much lower than the number of pixels in the background (non-cracks), network training using such unbalanced data may lead to segmentation results that are heavily biased towards high precision and low recall. For bridge crack detection, false negatives are more intolerable than false positives. Therefore, in order to alleviate the imbalance between crack and background (non-crack) pixels in bridge crack images and to achieve a better trade-off between precision and recall, this paper adopts a weighted combination of the balanced weighted cross-entropy loss, which has been used in the RCF network [48], and the Tversky loss [49] as the training loss function for the proposed network.

Let *P* and *G* denote the predicted segmentation result and the ground truth binary labels, respectively, and N is the total number of pixels. Then, the balanced weighted cross-entropy loss can be given by the following Equation (5):

(5) $$L_{BWCE}(W)=-\frac{1}{N}\sum_{i=1}^{N}\left(\alpha \cdot ((1-G)⊙\log(1-P))+\beta \cdot (G⊙\log(P))\right),$$

in which

(6) $$\begin{array}{c} \alpha =\lambda \cdot \frac{\left|Y^{+}\right|}{\left|Y^{+}\right|+\left|Y^{-}\right|}\\ \beta =\frac{\left|Y^{-}\right|}{\left|Y^{+}\right|+\left|Y^{-}\right|} \end{array},$$

where $\left|Y^{+}\right|$ and $\left|Y^{-}\right|$ represent the number of positive and negative samples, respectively, and the hyperparameter λ is the loss ratio to balance the positive and negative samples. W is the weight of the network model.

The Tversky loss is shown in Equation (7):

(7) $$L_{Tversky}(W)=\frac{\left|P\cap G\right|}{\left|P\cap G\right|+\alpha_{1}\left|P-G\right|+\beta_{1}\left|G-P\right|},$$

where |P−G| and |G−P| denote the total number of false positives and false negatives, respectively, and $\alpha_{1}$ and $\beta_{1}$ are hyper-parameters that control the trade-off between false positives and false negatives, affecting both recall and precision. Larger $\beta_{1}$ values weigh recall higher than precision. Therefore, we set $\beta_{1}=0.7$ and $\alpha_{1}=0.3$ to improve the performance of unbalanced data, which effectively reduces precision and improves recall.

By weighing the above two losses, the total loss is obtained as in Equation (8):

(8) $$L(W)=\eta \cdot L_{BWCE}(W)+(1-\eta )\cdot L_{Tversky}(W),$$

where η denotes the weight of loss $L_{BWCE}(W)$. The side outputs of each stage are reweighed in the training process, increasing the weights on the fusion side. The final total loss function is shown in Equation (9):

(9) $$L_{total}(W)=\sum_{k=1}^{5}w_{side}^{k}L(W)+w_{fuse}L(W),$$

where $w_{side}^{k},k\in \{1,2,3,4,5\}$ denotes the loss weight of the *k*th stage, and $w_{fuse}$ is the loss weight of the final fusion stage.

## 4. Experiments

### 4.1. Experimental Setup

The proposed network is based on the Pycharm 2021.2.1 software platform and is implemented using the open source framework PyTorch. The experiments were implemented on a NVIDIA RTX 2080Ti GPU with 8 G of RAM. The proposed network uses the Adam optimizer for parameter updating. In this study, the parameter beta1 was set to 0.5 and beta2 to 0.999. The initial learning rate was set to 1 × 10^−4^, the batch size to 1, and the number of training iterations to 500. A StepLR learning rate decay strategy was used, where the learning rate was decayed to 1/10 of the original rate for every 50 epochs. Data augmentation methods such as random rotation, horizontal flipping, rescaling, and Gaussian blurring were used to expand the training data and improve the generalization performance of the model.

### 4.2. Datasets

The network proposed in this paper was trained and evaluated using the publicly available bridge crack dataset BlurredCrack [2]. To further validate the adaptability of the model, two publicly available pavement crack datasets, CrackLS315 [20] and CFD [9], were used to verify the generalization ability of the network in this paper. The BlurredCrack, CrackLS315, and CFD datasets contain 2350, 315, and 118 crack images, respectively. For the BlurredCrack dataset, 1880 crack images were used for training and the remaining 470 for testing. For the CrackLS315 dataset, 275 crack images were used for training and the remaining 40 for testing. For the CFD dataset, 90 crack images were used for training and the remaining 28 for testing.

BlurredCrack: This dataset contains five sub-datasets collected from 10 bridges in Hunan and Guangdong provinces of China, totaling 189 high-resolution blurry crack images with 5120 × 5120 pixels, where the cracks mainly come from the surfaces of abutments, piers, and box girders in concrete bridge structures. The performance of the proposed network was evaluated on three typical sub-datasets. Due to the limitation of computational resources, the high-resolution images were cropped to obtain a total of 2350 crack images with 512 × 512 pixels, of which 1880 were used for training the proposed network and the remaining 470 for testing.

CrackLS315: This dataset contains 315 fine-grained images of pavement cracks, which were captured using a line-array camera under laser illumination. Each image has 512 × 512 pixels. The dataset was divided into training and test sets, with 275 selected as the training set and the rest 40 as the test set.

CFD: This is a publicly available pavement crack dataset widely used for crack detection. It contains 118 crack images with a resolution of 480 × 320. The images were resized to 512 × 512, and the output predicted segmentation image was adjusted to 480 × 320 for evaluating crack segmentation precision. The dataset was divided into 90 for training and 28 for testing.

### 4.3. Evaluation Metrics

Due to a significant category imbalance in the crack detection task, where the number of non-cracked samples greatly exceeds the number of cracked samples, accuracy alone may not accurately reflect the model’s performance. This is because it is insensitive to false predictions of non-cracks. To address this issue, the model’s performance was measured using recall and precision. Recall measures the model’s ability to correctly identify all cracks, while precision measures the proportion of predicted cracks that are actually cracks. In crack detection, it is crucial to ensure high recall to recognize all potential cracks, even if it means accepting some false positives with low precision. Therefore, using recall and precision better reflects the model’s practical application. To comprehensively evaluate the detection performance of the proposed network, four commonly used evaluation metrics were used, i.e., precision (Pr), recall (Re), F1 score, and intersection over union (IoU), to measure the performance of the proposed network for crack segmentation. Precision is defined as the ratio between the number of pixels correctly predicted to be cracks and the number of pixels predicted to be cracks, and it is given by Equation (10):

(10) $$\mathrm{Pr}=\frac{TP}{TP+FP}$$

Recall is defined as the ratio between the number of pixels correctly predicted to be cracked and the number of pixels in the ground truth that are cracked, and it is given by Equation (11):

(11) Re=TPTP+FN

The *F*1 score, which is a metric that takes both precision and recall into account, gives a balance between the two, as shown in Equation (12):

(12) F1=2⋅Pr⋅RePr+Re

*IoU* is a frequently used metric to measure the segmentation effect. In crack segmentation, *IoU* denotes the intersection ratio of the crack segmentation result and the ground truth of crack regions, which is given by Equation (13):

(13) $$IoU=\frac{TP}{TP+FP+FN}$$

In the above equation, *TP* denotes the number of pixels whose pixels are correctly predicted to be cracked, *FP* denotes the number of non-cracked pixels predicted as cracked pixels, and *FN* denotes the number of cracked pixels incorrectly predicted as non-cracked pixels.

### 4.4. Comparison with the State-of-the-Art (SOTA) Methods

This section compares the performances of MFSA-Net on three datasets with several state-of-the-art crack detection methods, including U-Net [16], RCF [48], DeepCrack [20], HDCBNet [2], and CrackFormer [23].

#### 4.4.1. The Results on BlurredCrack

**Figure 4:**
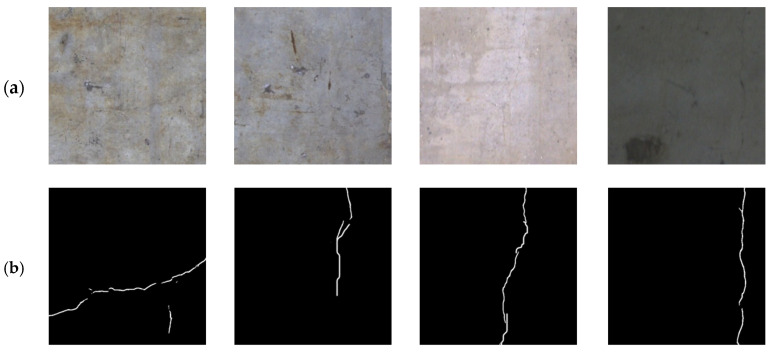

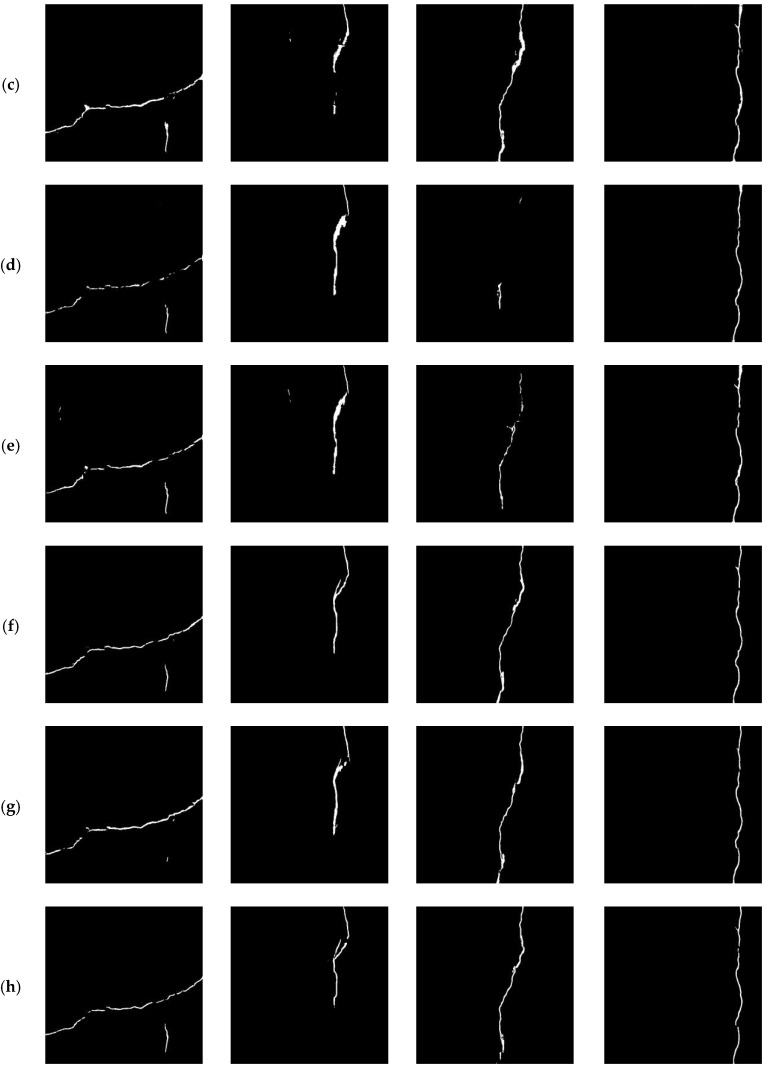
Comparison of different segmentation methods on the Bridge88 sub-dataset. (**a**) Original image; (**b**) ground truth; (**c**) U-Net [16]; (**d**) RCF [48]; (**e**) DeepCrack [20]; (**f**) CrackFormer [23]; (**g**) HDCBNet [2]; (**h**) MFSA-Net.

**Figure 5:**
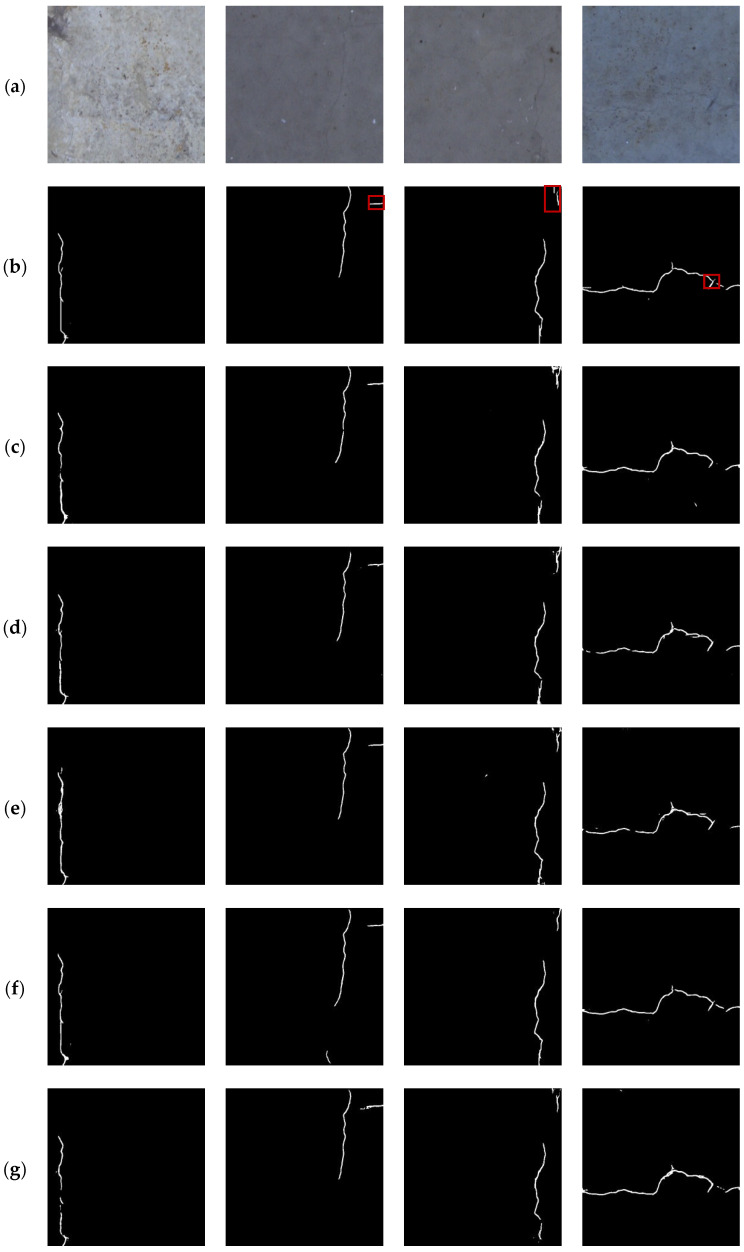

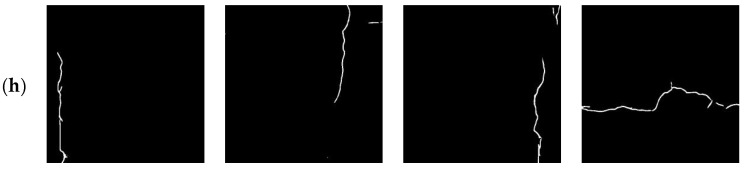
Comparison of different segmentation methods on the BridgeTL58 sub-dataset. (**a**) Original image; (**b**) ground truth; (**c**) U-Net [16]; (**d**) RCF [48]; (**e**) DeepCrack [20]; (**f**) CrackFormer [23]; (**g**) HDCBNet [2]; (**h**) MFSA-Net.

**Figure 6:**
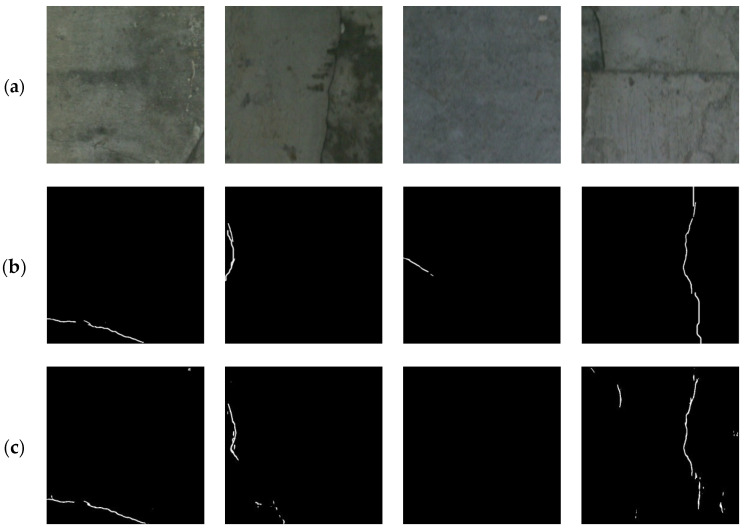

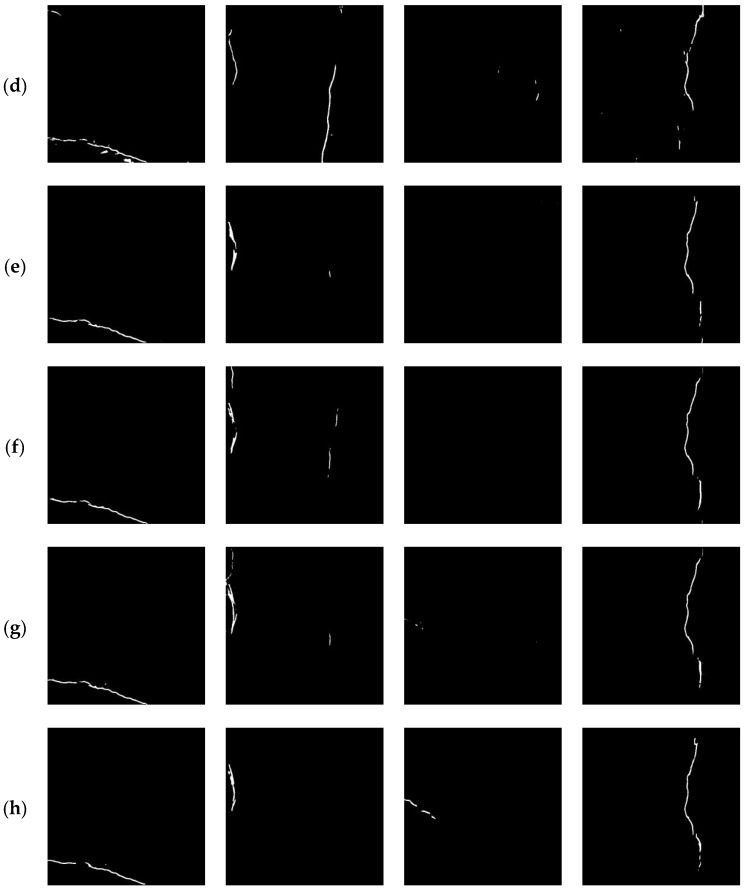
Comparison of different segmentation methods on the BridgeDB288 sub-dataset. (**a**) Original image; (**b**) ground truth; (**c**) U-Net [16]; (**d**) RCF [48]; (**e**) DeepCrack [20]; (**f**) CrackFormer [23]; (**g**) HDCBNet [2]; (**h**) MFSA-Net.

For the BlurredCrack dataset, experiments were conducted on three sub-datasets, namely, Bridge88, BridgeTL58, and BridgeDB288, and the visual comparisons of the proposed method with other crack segmentation methods are shown in Figure 4, Figure 5, and Figure 6, respectively.

From Figure 4, Figure 5 and Figure 6, it can be seen that the proposed method was able to detect the slender cracks well and also the local details of cracks, especially the tiny cracks that could not be detected by other methods. As shown in Figure 4, U-Net [16] segmented the cracks incompletely (column 2) and imprecisely (columns 1 and 3), RCF [48] barely segmented the cracks as shown in column 3, DeepCrack [20] showed discontinuous segmentation results (columns 1 and 3) with a lot of noise (columns 1 and 2), CrackFormer [23] was not precise enough to segment the details (column 2), and HDCBNet [2] lost small cracks (column 1). In contrast, MFSA-Net could segment different kinds of cracks more precisely. As shown in Figure 5, by observing the segmentation details marked in columns 2, 3, and 4 in Figure 5b, it can be seen that our method segmented the details more precisely, while the other methods missed them or showed false detection results. In particular, in column 3 of Figure 6, it can be observed that the cracks that could not be detected by other methods could still be detected by the proposed method.

The objective performance metrics are shown in Table 1. As can be seen from the comparison with other methods, MFSA-Net strikes a good balance between precision and recall. Specifically, MFSA-Net achieved the best performance on the BridgeTL58 and BridgeDB288 datasets. The F1 and IoU values on BridgeTL58 were 2.24% and 2.66% higher than the second-best result on BridgeDB288 of 6.04% and 7.78%, respectively. The best Pr was achieved on Bridge88, with the second-best F1 and IoU.

#### 4.4.2. The Results on CrackLS315

**Figure 7:**
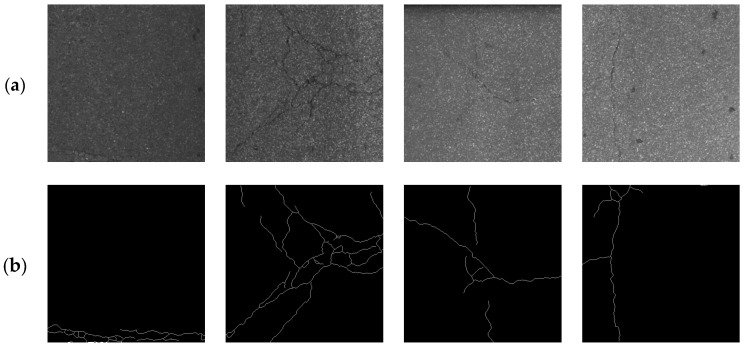

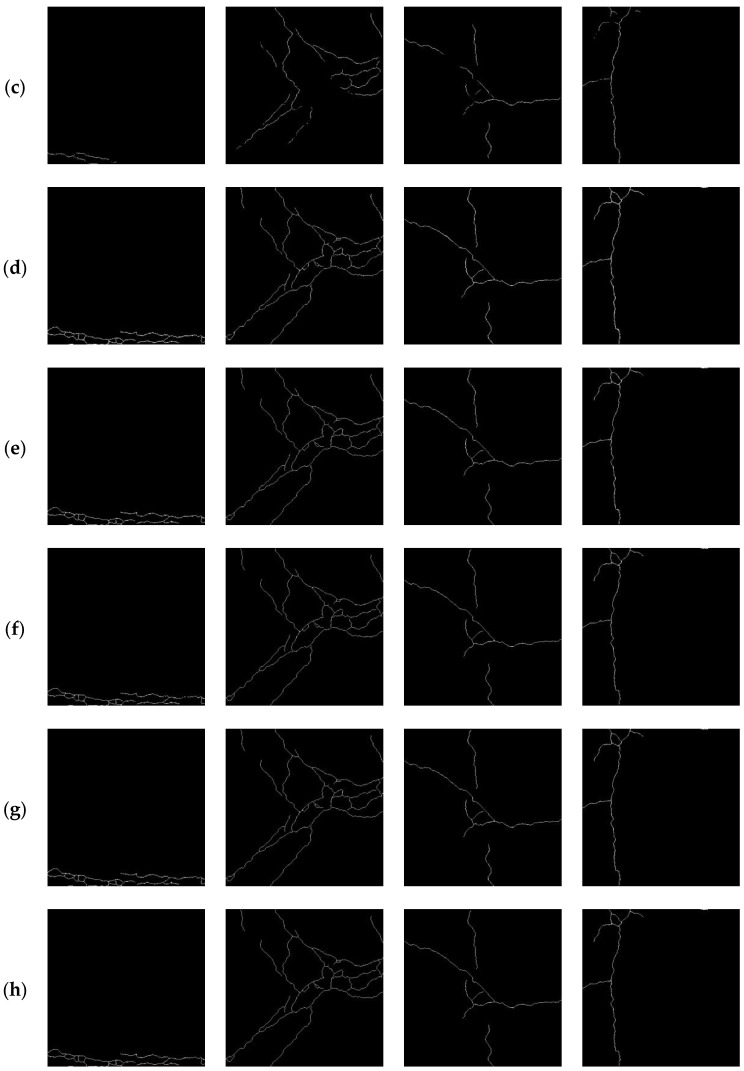
Comparison of different segmentation methods on the CrackLS315 dataset. (**a**) Original image; (**b**) ground truth; (**c**) U-Net [16]; (**d**) RCF [48]; (**e**) DeepCrack [20]; (**f**) CrackFormer [23]; (**g**) HDCBNet [2]; (**h**) MFSA-Net.

The challenge with this dataset is that the images have extremely low contrast. The detection results are given in Table 2, where it can be seen that MFSA-Net achieved optimal performance on all evaluation metrics on the CrackLS315 dataset. This indicates that the proposed network is not only adaptable to the detection of bridge cracks in complex backgrounds but also effective enough to detect road cracks. Compared with the suboptimal CrackFormer [19], it obtained a gain of 4.98% on Pr, 1.59% on Re, 3.68% on F1, and 5.95% on IoU, respectively. The F1 and IoU metrics of U-Net [16], RCF [48], DeepCrack [20], and HDCBNet [2] were 23.30% and 31.77%, 12.68% and 18.77%, 5.21% and 8.22%, and 3.73% and 5.95% lower than MFSA-Net, respectively. As can be seen from the visualization results in Figure 7, MFSA-Net could detect more detailed and complex thin cracks on low-contrast pavements with more accurate and complete results.

#### 4.4.3. The Results on CFD

**Figure 8:**
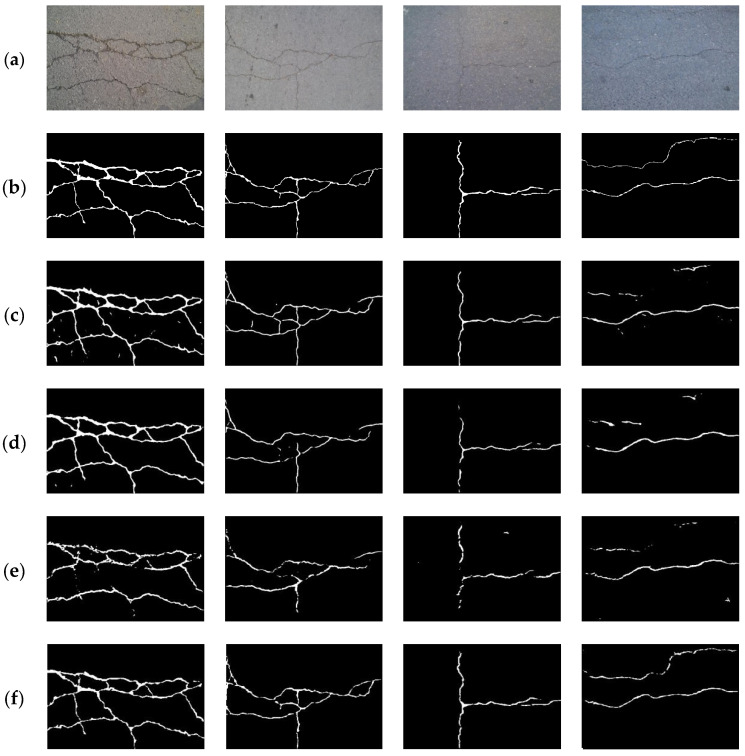

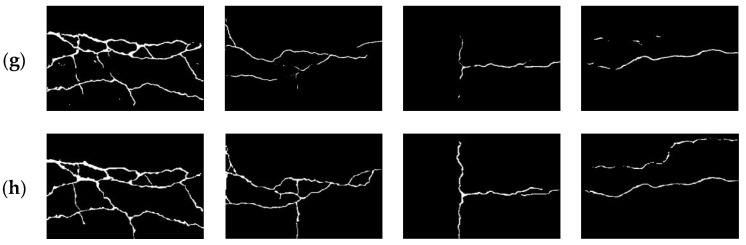
Comparison of different segmentation methods on the CFD dataset. (**a**) Original image; (**b**) ground truth; (**c**) U-Net [16]; (**d**) RCF [48]; (**e**) DeepCrack [20]; (**f**) CrackFormer [23]; (**g**) HDCBNet [2]; (**h**) MFSA-Net.

The CFD dataset is a popular public dataset for pavement crack detection, for which quantitative and qualitative comparison analysis with crack segmentation methods was performed. It can be seen from the quantitative evaluation indicators in Table 3 that the proposed method achieved the best results in all the evaluation indexes, which means the proposed method is obviously better than other methods. Figure 8 shows some of the segmentation results of the proposed method and other crack segmentation methods. From the visual effect, it can be seen that the proposed method could precisely detect all crack defects and suppress a lot of background interference information, while other methods were affected by the interference information and had a lot of noise in the detection results.

### 4.5. Ablation Study

As a further check on the gain of each module in the proposed model, the ablation study was performed on the Bridge88 dataset.

#### 4.5.1. Verifying the Validity of the Strip Decoder

The ablation study was conducted to verify the effectiveness of the strip decoder while keeping the proposed encoder fixed. The experimental results are shown in Table 4.

Table 4 shows the ablation experiments on the decoder in Figure 1. As can be seen from Table 4 (row 2), the decoder built using the 3 × 3 convolution had the worst performance in all metrics. Table 4 (row 3) indicates that the decoder built using the self-attention module in Crackformer [23] performed slightly better than the SCM decoder in terms of Pr, but in all other metrics, the performance was lower than the SCM decoder, thus validating the effectiveness of the SCM decoder. The reason is that the SCM decoder is able to acquire global and local information, while the 3 × 3 convolution decoder can only obtain local information, which is poor in detecting long and thin cracks, and the self-attention decoder only focuses on global information and ignores the local detail features, which is weak in the detection of short cracks.

#### 4.5.2. Impact of the SCM’s Position in the Encoder on the Results

At different stages of the encoder, conventional square convolution and strip convolution are employed to construct the encoder. The proper use of strip convolution in the encoder has a large impact on the results of the detection. In this experiment, the basic SegNet encoder was used as a baseline (row 1 of Table 5) and considered in four scenarios, as shown in Table 5. First, using SCM in the last building block of each stage, we obtained 73.37% on the F1 score (row 2 of Table 5). Secondly, we tried to use SCM in all the building blocks of the last stage and obtained 73.12% on the F1 score (row 3 of Table 5), with a slight decrease in performance. Next, when SCM was employed in the last building block of the last three stages, an F1 score of 76.28% was yielded (row 4 of Table 5). However, when trying to use SCM for all the building blocks of the encoder, there was nearly no performance gain (row 5 of Table 5). The above results illustrate that using SCM in the last building block of the last three stages of the encoder can improve the segmentation performance of the network.

## 5. Conclusions

This paper focuses on concrete bridge cracks, which are characterized by long and narrow spans, and proposes MFSA-Net, a pixel-level concrete bridge crack detection network with multi-stage feature aggregation and structure-aware convolutional blocks that realizes the structure awareness of concrete bridge cracks by interactively combining square convolution and strip convolution. The proposed network was trained and tested on the publicly available concrete bridge crack dataset BlurredCrack, and the average results of the proposed method on the evaluation metrics were 73.74%, 77.04%, 75.30%, and 60.48% for precision, recall, F1 score, and IoU, respectively, which are satisfactory results. At the same time, MFSA-Net was found to be capable of detecting clearer crack boundaries as well as local details of the cracks. The proposed method was tested on the concrete pavement crack datasets CrackLS315 and CFD, and the proposed method achieved satisfactory results in all evaluation metrics on both CrackLS315 and CFD datasets with precision of 82.37% and 88.60%, recall of 99.10% and 85.61, F1 score of 89.97% and 87.08, and IoU of 81.76% and 77.12%. Meanwhile, the proposed method has obvious advantages in the detection of slender and tiny cracks. The experimental results show that the proposed method has significant generalization ability and ensures robustness of detection.

According to the research methods and results, the following conclusions can be drawn: (1) Both local detail features and global semantic features are very important to the crack segmentation of concrete bridges. Different from the existing methods for detecting thin cracks, which mainly obtain global features by increasing the receptive field, the proposed MFSA-Net mainly combines the advantages of square convolution and strip convolution, which can not only increase the receptive field to obtain global features but also suppress the background interference information brought by it, which helps detect thin and long cracks. (2) Effective feature fusion methods can enhance crack features. The feature attention fusion module designed in this study can fuse local and global features to enhance the feature representation ability of cracks. At the same time, the module is embedded in different stages of MFSA-Net to gradually refine the crack segmentation results and improve crack detection precision. (3) The method has a reasonable loss function design. In bridge crack detection, there is a serious imbalance between foreground (crack) and background (non-crack), which easily leads to network bias, and the segmentation results are heavily biased towards high precision and low recall. Tversky loss was used in this study, which can adjust this imbalance bias according to the ratio of foreground and background in the sample so that the prediction results are in line with expectations.

However, there are still some shortcomings in this study. Some cracks still could not be accurately detected when subjected to severe interference, and some crack discontinuities were also observed. In the future, a priori information about cracks should be considered being added to the network to improve the precision of detection. Secondly, this research was focused on the segmentation of cracks in concrete bridges. Future studies should expand this to include surface crack detection in other materials (e.g., steel or composites, etc.) as well as other structures (e.g., houses, tunnels, dams, etc.) to broaden the scope of the proposed network and enhance its applicability in the real world. In addition, this study focused on the detection of bridge crack images without considering the crack depth problem that exists in practice. In the future, research can focus on the 3D reconstruction of cracks, which is more in line with the actual engineering needs, which is also a hot research topic at present.

## Figures and Tables

**Table 1:** Comparison of different methods in three bridge sub-datasets.

Methods	Bridge88				BridgeTL58				BridgeDB288			
	Pr (%)	Re (%)	F1 (%)	IoU (%)	Pr (%)	Re (%)	F1 (%)	IoU (%)	Pr (%)	Re (%)	F1 (%)	IoU (%)
U-Net [16]	58.01	77.80	66.46	49.77	71.43	66.71	68.99	52.66	66.30	61.46	63.79	46.83
RCF [48]	60.38	69.50	64.62	47.74	62.87	62.79	62.83	45.80	64.54	58.48	61.36	47.13
DeepCrack [20]	57.69	72.67	64.32	47.40	61.10	62.68	61.88	48.66	61.03	56.78	58.83	47.47
CrackFormer [23]	71.07	86.78	78.14	64.13	63.10	71.81	67.17	50.57	73.68	71.09	72.36	56.69
HDCBNet [2]	72.47	64.58	68.30	51.86	66.81	60.25	63.36	46.37	60.42	63.54	61.94	50.40
MFSA-Net	76.21	76.35	76.28	61.65	67.17	75.81	71.23	55.32	77.83	78.97	78.40	64.47

**Table 2:** Comparison of different methods on the CrackLS315 dataset.

Methods	Pr (%)	Re (%)	F1 (%)	IoU (%)
U-Net [16]	65.33	68.05	66.67	49.99
RCF [48]	69.52	87.02	77.29	62.99
DeepCrack [20]	74.66	98.00	84.76	73.54
CrackFormer [23]	77.39	97.51	86.29	75.89
HDCBNet [2]	76.79	98.34	86.24	75.81
MFSA-Net	82.37	99.10	89.97	81.76

**Table 3:** Comparison of different methods on the CFD dataset.

Methods	Pr (%)	Re (%)	F1 (%)	IoU (%)
U-Net [16]	71.82	75.97	73.83	58.52
RCF [48]	67.00	70.86	68.88	52.53
DeepCrack [20]	60.42	66.52	63.32	46.33
CrackFormer [23]	83.57	83.93	83.75	72.04
HDCBNet [2]	66.70	61.29	63.88	46.93
MFSA-Net	88.60	85.61	87.08	77.12

**Table 4:** Ablation study on the decoder. Square convolution: a 3 × 3 convolutional block was used to build the decoder. Self-attention: a self-attention block was used to build the decoder. SCM: the strip convolutional module was used to build the decoder.

Decoder	Pr (%)	Re (%)	F1 (%)	IoU (%)
Square convolution	67.39	73.54	70.62	54.23
Self-attention	76.62	72.08	74.28	58.66
SCM	76.21	76.35	76.28	61.65

**Table 5:** Ablation analysis of SCM’s position. (L: last building block in each stage, A: all building blocks in the last stage, and LLT: last building block in the last three stages).

Encoder	SCM Position	Pr (%)	Re (%)	F1 (%)	IoU (%)
Base SegNet	-	66.89	70.44	68.62	52.07
Base SegNet + SCM	L	72.04	74.75	73.37	56.78
Base SegNet + SCM	A	71.96	74.32	73.12	56.14
Base SegNet + SCM	LLT	76.21	76.35	76.28	61.65
Base SegNet + SCM	A + L	66.92	70.70	68.76	52.41

## Data Availability

No new data were created or analyzed in this study. Data sharing is not applicable to this article.

## Glossary

**Symbols**	**Description**
ℝ	real number space
X	input tensor
$A_{mask}^{k}$	attention mask map of the *k*th stage
$S_{side}^{k}$	side output of the *k*th stage
Γ	tensor concatenation operation
σ	Sigmoid activation function
$Up_{H\times W}$	upsampling to the input image size H×W
$S_{fuse}$	Output prediction map after multi-stage fusion, i.e., the final prediction result map
⊗	convolution operation
⊕	element-wise addition operation
⊙	element-wise multiplication operation
P	the predicted segmentation result
G	the ground truth binary label
N	the total number of pixels in a crack image
α	predicting the error weights
β	predicting the correct weights
$\left|Y^{+}\right|$	the number of positive samples in a crack image
$\left|Y^{-}\right|$	the number of negative samples in a crack image
λ	the loss ratio to balance the positive and negative samples
$L_{BWCE}(W)$	the balanced weighted cross-entropy loss
$\alpha_{1}$	false positive weights
$\beta_{1}$	false negative weights
$L_{Tversky}(W)$	Tversky loss
L(W)	$L_{BWCE}(W)$ and $L_{Tversky}(W)$ weighted losses
η	the weight of loss $L_{BWCE}(W)$
$L_{total}(W)$	The final total loss function
$w_{side}^{k}$	the loss weight of the *k*th stage
$w_{fuse}$	the loss weight of the final fusion stage
∑	Summation
